# Cellular Cholesterol Distribution Influences Proteolytic Release of the LRP-1 Ectodomain

**DOI:** 10.3389/fphar.2016.00025

**Published:** 2016-02-12

**Authors:** Bassil Dekky, Amandine Wahart, Hervé Sartelet, Michaël Féré, Jean-François Angiboust, Stéphane Dedieu, Olivier Piot, Jérôme Devy, Hervé Emonard

**Affiliations:** ^1^Laboratoire de Signalisation et Récepteurs Matriciels, UFR de Sciences Exactes et Naturelles, Université de Reims Champagne-ArdenneReims, France; ^2^CNRS, Matrice Extracellulaire et Dynamique Cellulaire, UMR 7369Reims, France; ^3^MéDIAN-Biophotonique et Technologies pour la Santé, UFR de Pharmacie, Université de Reims Champagne-ArdenneReims, France; ^4^Plateforme d’Imagerie Cellulaire et Tissulaire, Université de Reims Champagne-ArdenneReims, France

**Keywords:** LRP-1, low-density lipoprotein receptor-related protein-1, ectodomain, cholesterol, shedding, Raman microspectroscopy

## Abstract

Low-density lipoprotein receptor-related protein-1 (LRP-1) is a multifunctional matricellular receptor composed of a large ligand-binding subunit (515-kDa α-chain) associated with a short trans-membrane subunit (85-kDa β-chain). LRP-1, which exhibits both endocytosis and cell signaling properties, plays a key role in tumor invasion by regulating the activity of proteinases such as matrix metalloproteinases (MMPs). LRP-1 is shed at the cell surface by proteinases such as membrane-type 1 MMP (MT1-MMP) and a disintegrin and metalloproteinase-12 (ADAM-12). Here, we show by using biophysical, biochemical, and cellular imaging approaches that efficient extraction of cell cholesterol and increased LRP-1 shedding occur in MDA-MB-231 breast cancer cells but not in MDA-MB-435 cells. Our data show that cholesterol is differently distributed in both cell lines; predominantly intracellularly for MDA-MB-231 cells and at the plasma membrane for MDA-MB-435 cells. This study highlights the relationship between the rate and cellular distribution of cholesterol and its impact on LRP-1 shedding modulation. Altogether, our data strongly suggest that the increase of LRP-1 shedding upon cholesterol depletion induces a higher accessibility of the sheddase substrate, i.e., LRP-1, at the cell surface rather than an increase of expression of the enzyme.

## Introduction

The low-density lipoprotein receptor-related protein-1 (LRP-1) is a large heterodimeric receptor composed of an heavy extracellular chain, the 515-kDa α-chain, non-covalently associated with a light transmembrane chain, the 85-kDa β-chain (Emonard et al., 2014). The extracellular α-chain exhibits four cystein-rich complement-type repeats which bind more than 40 ligands, including proteinases and proteinase:inhibitor complexes (Etique et al., 2013). Motifs of the intracellular part of the β-chain activate endocytosis and signaling pathways (Lillis et al., 2005), which drive numerous biological functions and play a key role in the development of many pathological disorders (Lillis et al., 2008; Van Gool et al., 2015). LRP-1 invalidation in mice is lethal at early stage of embryogenesis (Herz et al., 1992). We previously demonstrated that LRP-1 promotes invasion of malignant cells by modulating focal complex composition (Dedieu et al., 2008).

Low-density lipoprotein receptor-related protein-1 is broadly expressed in multiple cell types such as mesenchymal and epithelial cells (Emonard et al., 2014). Its expression is regulated by hormones and growth factors that induce different responses depending on cell types. Cell surface LRP-1 is cleaved by shedding to generate soluble LRP-1 ectodomain composed of the entire extracellular α-chain linked to the extracellular part of the β-chain which was first discovered in plasma (Quinn et al., 1997). The first LRP-1 sheddase was characterized in human choriocarcinoma BeWo cells as a metalloproteinase (Quinn et al., 1999). More recently our group identified a disintegrin and metalloproteinase-12 (ADAM-12) and membrane-type 1 matrix metalloproteinase (MT1-MMP; Selvais et al., 2009, 2011). Several proteolytic enzymes belonging to other proteinases families have also been identified (for a review, Emonard et al., 2014).

Shedding is a closely regulated process that controls most of types I and II transmembrane proteins levels at cell surface (Hartmann et al., 2013). Cellular cholesterol depletion stimulates shedding of the interleukin-6 receptor (Matthews et al., 2003) and CD30 antigen (von Tresckow et al., 2004). By comparing two cell lines exhibiting different levels of cholesterol (conventional human fibrosarcoma HT1080 cells and an epithelioid variant with a twofold higher cell cholesterol content), we previously showed that low cell cholesterol level promotes LRP-1 shedding (Selvais et al., 2011).

Cholesterol is widely expressed at cell surface of mammalian cells but can also be located in the cytosolic compartment where it could play a role in transmembrane protein trafficking (Mukherjee et al., 1998). In the present study, we evaluated the efficiency of LRP-1 shedding process in cell lines expressing either cholesterol at plasma membrane or in cytosolic compartment (Nieva et al., 2012). We demonstrated by using different imaging approaches, that efficient extraction of cholesterol and increased LRP-1 shedding occur predominantly in cells exhibiting cholesterol at cell surface.

## Materials and Methods

### Reagents and Antibodies

Dulbecco’s modified Eagle medium (DMEM) and other cell culture reagents were purchased from Thermo Fisher Scientific (Illkirch, France). Fetal calf serum (FCS) was from Dutscher (Brumath, France). Filipin, methyl-β-cyclodextrin (MβCD) and other chemicals were from Sigma-Aldrich (Saint-Quentin Fallavier, France). Phosphate-buffered saline (PBS)-B (131 mM NaCl, 5.1 mM Na_2_HPO_4_, and 1.5 mM KH_2_PO_4_) was from bioMérieux (Craponne, France). Anti-LRP-1 α-chain (mouse, clone 8G1) was from Calbiochem (Merck Biosciences, distributed by VWR International, Strasbourg, France). Goat polyclonal antibodies directed against β-actin were from Abcam (Paris, France). Horseradish peroxidase (HRP)-conjugated anti-mouse antibodies were from Cell Signaling Technology (distributed by Ozyme, Montigny-Le-Bretonneux, France) and HRP-anti-goat antibodies from Sigma-Aldrich.

### Cell Culture

Human breast cancer cell lines MDA-MB-231 and MDA-MB-435 were obtained from the American Type Culture Collection. MDA-MB-231 and MDA-MB-435 cells were cultured in DMEM containing 1 and 4.5 g/l glucose, respectively. Culture media were supplemented with 10% FCS, 100 units/ml penicillin and 10 mg/ml streptomycin. For cell imaging, FCS was depleted in lipoproteins following a procedure adapted from the Havel et al. (1955). Cells were grown at 37°C in a humid atmosphere (5% CO_2_ and 95% air). As cellular cholesterol content depends, at least in part, on cellular confluency state (Takahashi et al., 2007), all experiments were carried out at similar cell densities.

### MβCD Treatment and Cholesterol Assay

The water-soluble MβCD forms soluble inclusion complexes with cholesterol, enhancing thus its solubility in aqueous solution (Pitha et al., 1988) and is classically used to extract cholesterol from cultured cells. In the present study, cells were treated with MβCD (0–20 mM) in FCS-free medium for 30 min at 37°C. Cells were then harvested in reaction buffer (0.1 M potassium phosphate, pH 7.4, 50 mM NaCl, 5 mM cholic acid, and 0.1% Triton X-100) and sonicated. Cholesterol content was quantified using the Amplex Red cholesterol assay kit (Invitrogen distributed by Thermo Fisher Scientific), as recommended by the manufacturer. Reactions proceeded for 20 min at 37°C. Alternatively, after treatment with MβCD cells were washed with PBS and further incubated in FCS-free medium for 24 h. Twenty four-hour conditioned media were concentrated and shedding of LRP-1 was analyzed by western blotting.

### Raman Microspectroscopy Analysis

Cells (3 × 10^4^) were seeded in 6-well plates containing CaF_2_ substrates (Crystan, Ltd., Dorset, UK), and 48 h later cells were fixed with 4% cold paraformaldehyde (PFA) for 30 min at room temperature. After fixing, cells were washed three times with PBS and water, before drying to be analyzed with Raman spectroscopy.

Raman spectra were acquired from each sample using a LabRAM Raman spectrometer (Jobin Yvon, Horiba, Lille France). The setup contained a laser diode at 660 nm supplying an excitation beam of 25 mW at the sample. The laser beam was focused onto the sample using a Leica HCX PL FluoTar x100 objective (NA = 0.75). The same objective collected the light scattered from the sample. An edge filter permitted to reject the laser reflection and the Rayleigh scattering. A grating of 1200 g/mm ensured the dispersion of the Raman Stokes signal with a spectral resolution of 4 cm^-1^. The intensity of the Raman vibrations was measured using a deep depletion charge-coupled device (CCD) detector. The spectra were collected on a total spectral range from 400 to 4000 cm^-1^, with an acquisition time of 40 s per spectrum. For each cell, a number of five spectra were collected at the level of the cytoplasm. Raman data were then baseline-corrected using linear segments, slightly smoothed (three points averaging) and normalized on the basis of the total integrated intensity. The five spectra recorded on each cell were averaged. Up to this point, the acquisition and processing of the data were performed using Labspec 5 software (Horiba Jobin Yvon, Lille France). Then, Raman data were submitted to statistical multivariate processing corresponding to principal component analysis (PCA). PCA operated via a home-made interface using Matlab Toolbox (MathWorks^®^). Mean-centered data were used for PCA.

### Cholesterol Staining and Cell Imaging

Cells (5 × 10^4^) were seeded onto gelatin-coated glass slides and cultured in media containing lipoproteins-depleted FCS for 24 h at 37°C. Then, cells were treated or not with MβCD and fixed in 3% paraformaldehyde for 60 min at room temperature. After three washes in PBS, cells were incubated in glycine (1.5 mg/ml in PBS-B) for 10 min and then stained with filipin (0.05 mg/ml in PBS-B) for 2 h at room temperature. Filipin-stained cell preparations were analyzed using a Zeiss LSM 710 confocal laser scanning microscope with the 63x oil-immersion objective and Zen operating system (Zeiss, Heidelberg, Germany). All acquisitions were performed with UPlan x 63, 1.4 numerical aperture objective by exciting filipin with a chameleon infrared laser tuned at 740 nm. Emitted fluorescence was detected through the appropriate filter and twenty images were captured with a 0.25-μm z-step. DIC images were acquired simultaneously with the reflected light images using a TPMT module. Images were treated with Amira^TM^ software (v6.0.1, FEI visualization Sciences Group, Merignac, France) and projection through each z-stack was merged with DIC images.

### RNA Isolation, RT-PCR, and Real-Time-PCR

Total RNAs were isolated and purified with Extract-All kit (Eurobio Laboratories, Courtaboeuf, France). Reverse transcription (RT) and real-time PCR were performed with Verso SYBR 2-Step QRT Rox kit (AB-4113/A) and Absolute QPCR SYBR Green Rox (AB-1162/B), respectively (Thermo Fisher Scientific). Quantitative PCR was carried out on a Chromo4 Real-Time Detector (Bio-Rad Laboratories, Marne-la-Vallée, France). Data were normalized to ribosomal proteins L32 (RPL32) and S18 (RS18) or to β-actin. Primers for LRP-1 (Dedieu et al., 2008) and β-actin (Langlois et al., 2010) were previously described. Primers were synthesized as follows: for MT1-MMP, AACCAAGTGATGGATGGATACC and CTCCTTAATGTGCTTGGGGTAG; for transmembrane form of ADAM-12, ADAM-12L, GGGCTGTAGCTGTCAAATGG and CTGACTTCCGGCAGGTTAAA; for RPL32, CATTGGTTATGGAAGCAACAAA and TTCTTGGAGGAAACATTGTGAG; for RS18, GCAGAATCCACGCCAGTACAA and GCCAGTGGTCTTGGTGTGCT (Eurogentec France, Angers, France). Results shown are representative of three independent experiments.

### Sample Preparation and Western Blot Analysis

Cells treated or not with MβCD were then washed with PBS and further incubated with FCS-free medium for 24 h. Twenty four-hour-conditioned media were cleared by centrifugation (1,000 *g* at 4°C for 10 min) and concentrated 50-fold with Vivaspin centrifugal concentrators (Sartorius Stedim Biotech, distributed by Dutscher) following manufacturer’s recommendations. Cells were scraped in ice-cold lysis buffer (10 mM CHAPS, 20 mM HEPES (pH 7.4), 150 mM NaCl, 2 mM CaCl_2_, 1 mM phenylmethylsulfonylfluroide supplemented with proteinase inhibitor cocktail from Sigma-Aldrich). After sonication, the remaining pellet was discarded by centrifugation (10,000 *g* at 4°C for 10 min).

Western blotting was performed as previously described (Selvais et al., 2011) using 5% polyacrylamide gel for LRP-1 515 kDa α-chain and 10% polyacrylamide gel for β-actin. Primary antibodies were used at 1/4000 for LRP-1 α-chain and 1/1000 for β-actin. Samples were normalized with respect to cell protein amount, which was determined using BC assay protein quantitation kit (Thermo Scientific, distributed by Interchim, Montluçon, France). Each lane was loaded with cell lysates equivalent of 40 μg protein, or corresponding amounts of conditioned medium. Immunoreactive bands were revealed using the ECL chemiluminescence kit (Amersham Biosciences, distributed by Dutscher), acquired using the Odyssey^®^ Fc Dual-Mode LI-CORE Imaging System (Biosciences Biotechnology, distributed by Eurobio Laboratories) and quantified using ImageJ software. β-actin antibodies were used for normalization. Immunoblots presented are representative of at least three independent experiments.

### Statistical Analysis

Data were analyzed using unpaired two-tailed Student’s *t*-test. Differences were considered significant for *P* < 0.05. Values are reported as mean ± SD.

## Results

We previously reported in the human fibrosarcoma HT1080 cell line the correlation between cell cholesterol amount and efficiency of LRP-1 shedding (Selvais et al., 2011). In the present study we investigated the possible role of cholesterol distribution by using two different breast cancer cell lines MDA-MB-231 and MDA-MB-435 cells, one expressing cholesterol in the cytosol and the other at the plasma membrane (Nieva et al., 2012).

### MDA-MB-231 and MDA-MB-435 Breast Cancer Cells Exhibit Different Lipid Phenotypes

**FIGURE 1:**
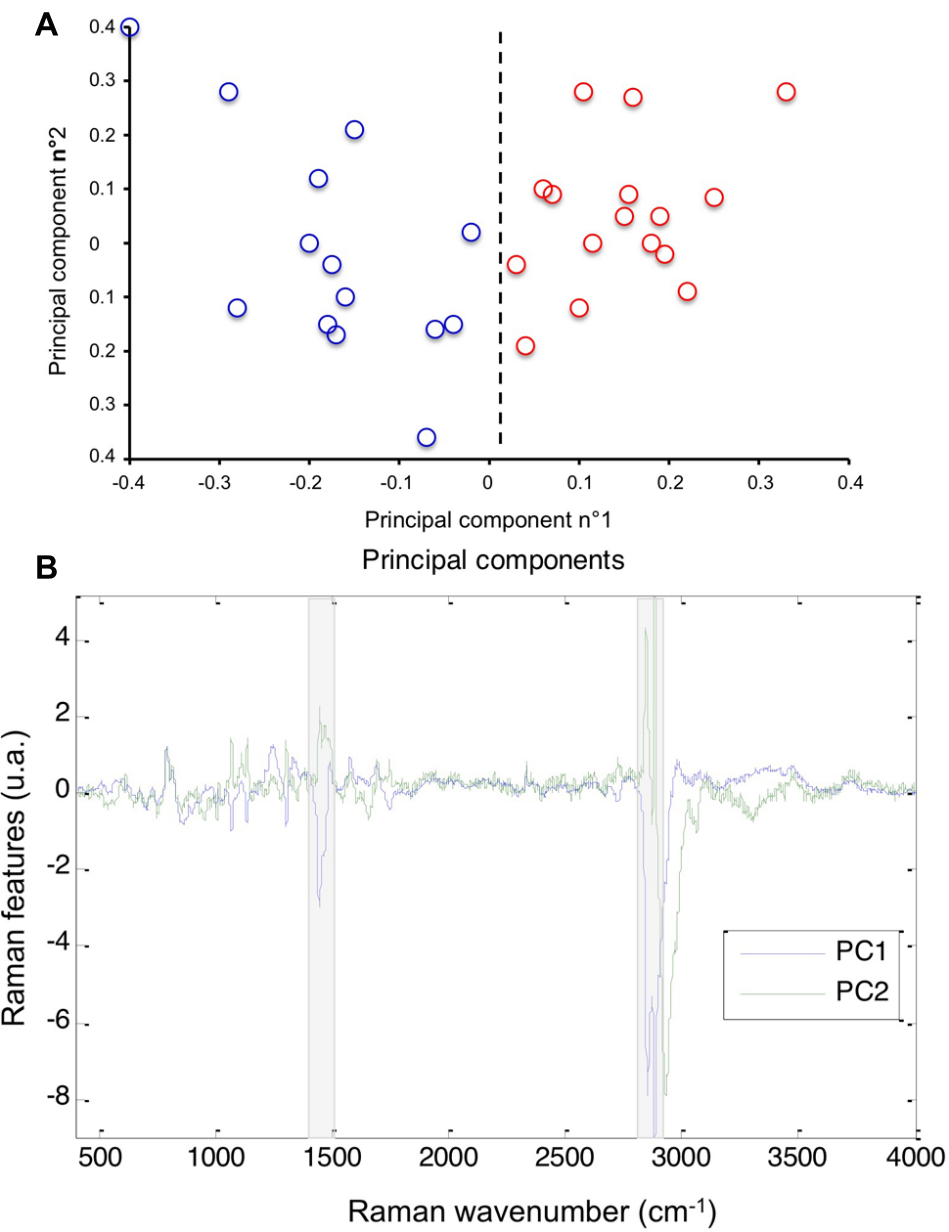
**Cytoplasmic lipid profiling of MDA-MB-231 and MDA-MB-435 cells by Raman analysis. (A)** Score plot resulting of the PCA processing of Raman spectra collected on MDA-MB-231 (red circles) and MDA-MB-435 (blue circles) cells. The scores were projected on the two first principal components. **(B)** Display of the two first principal components. The Raman features comprising the main variability of the spectral data, were highlighted by the grayed underlining.

We first explored the lipid phenotype of MDA-MB-231 and MDA-MB-435 cells by Raman microspectroscopy. Spectra collected on the cytoplasmic compartment of the two cell lines were processed by PCA, an exploratory unsupervised method of multivariate data processing. PCA is commonly used to explore the intra- and inter-group variabilities based on the Raman signals of the cells (Poplineau et al., 2011). A distinction between the two cell types is clearly visible on the score plot constructed on the two first components (**Figure 1A**). The distinction relies mainly on the first principal component (PC1) that exhibits signals assigned to lipid vibrations (**Figure 1B**). Indeed, the spectral zones grayed on the display of the first component, centered at 1450 and 2850 cm^-1^, are assigned to bending and stretching vibrations of lipid CH_2_ and CH_3_ groups respectively. This analysis reflects that the lipid contribution of the cytoplasm as probed by Raman spectroscopy allows distinguishing MDA-MB-231 and MDA-MB-435 cells. The same Raman features are also recovered on the second principal component (PC2), reflecting their involvement in the intra-group variability of these cellular samples.

### Efficiency of Cholesterol Depletion Depends on its Cellular Distribution

We next investigated using biochemical and cellular imaging analyses whether the biophysical analysis findings showing different cytoplasmic lipids-based discrimination between MDA-MB-231 and MDA-MB-435 cells was confirmed for cholesterol.

**FIGURE 2:**
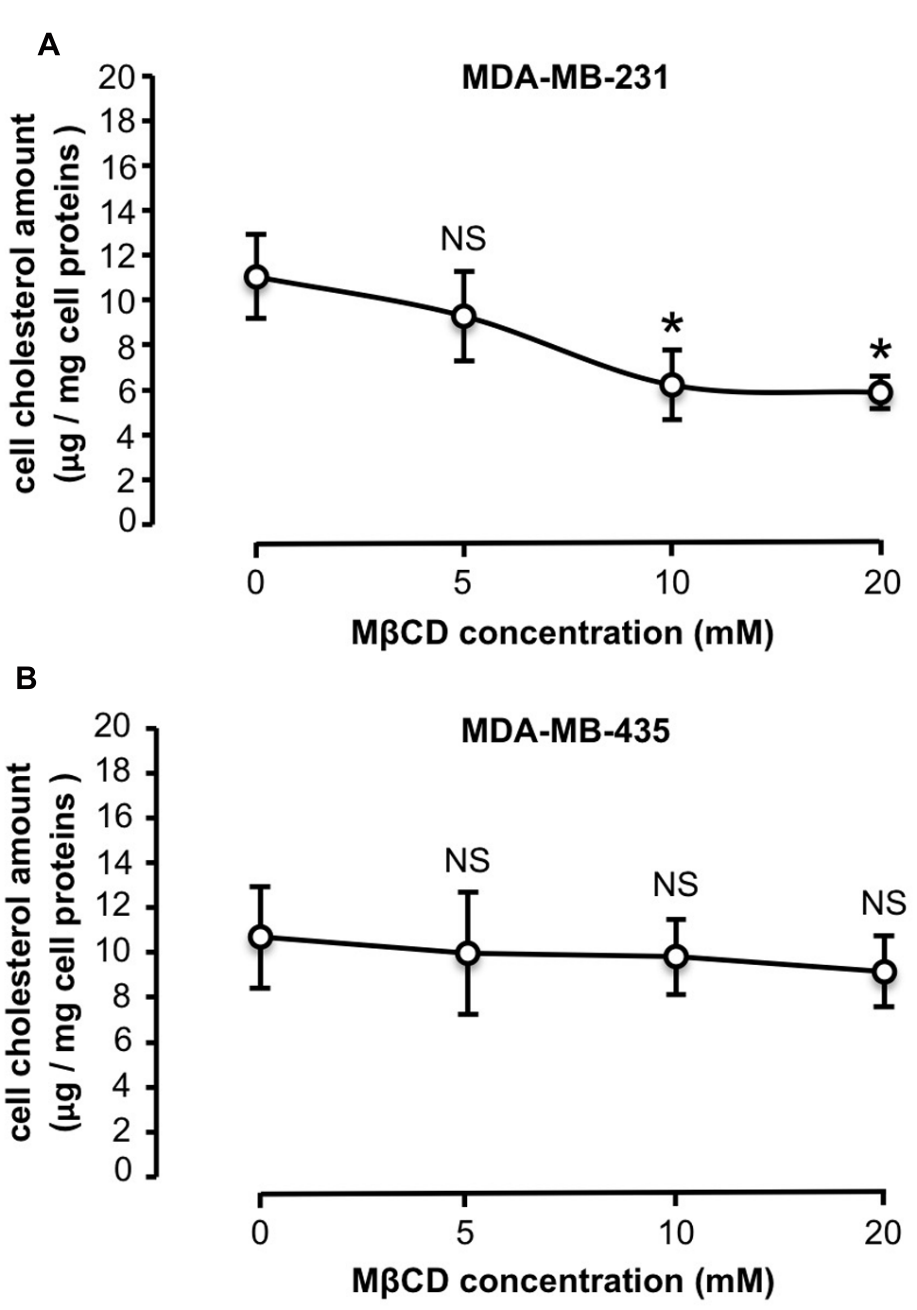
**Differential efficiency of MβCD for depleting cholesterol.** MDA-MB-231 **(A)** and MDA-MB-435 **(B)** breast cancer cells were treated as described under Section “Materials and Methods” with increasing concentrations of MβCD and cellular cholesterol content was then measured. Values expressed as μg cholesterol/mg cell protein are mean ± SD (*n* = 6 for each cell line). NS, not significant, ^∗^*P* < 0.05; Student’s *t*-test.

**FIGURE 3:**
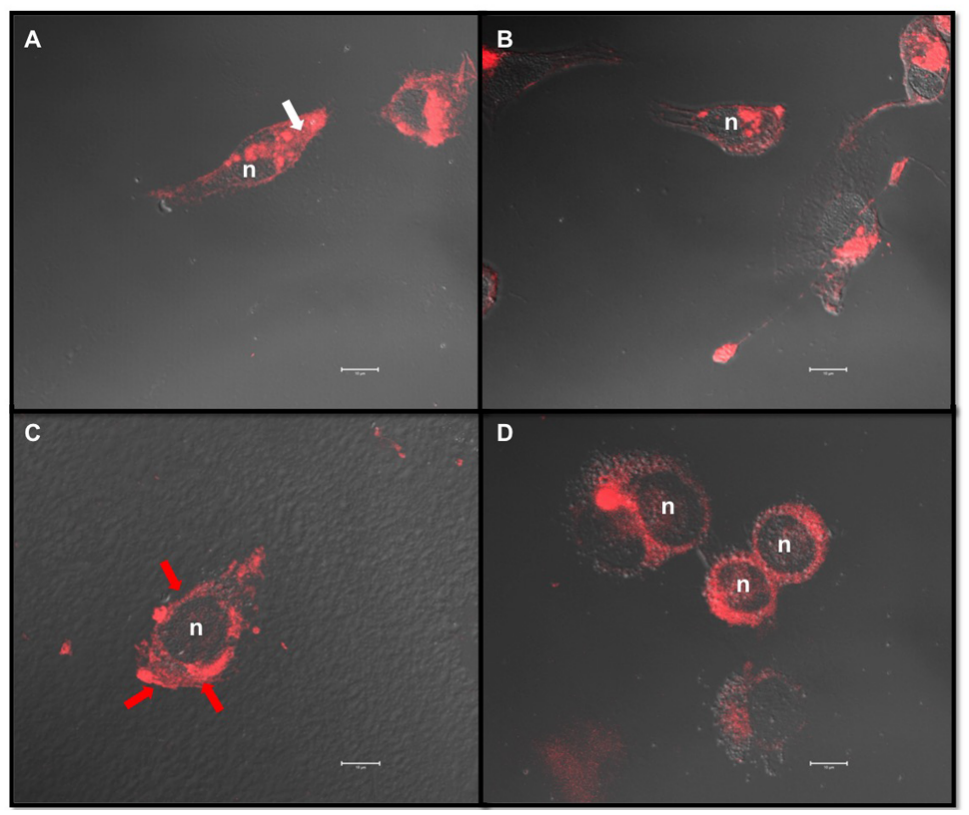
**MβCD exhibits different effects on cholesterol depletion in MDA-MB-231 and MDA-MB-435 breast cancer cell lines.** MDA-MB-231 **(A,B)** and MDA-MB-435 **(C,D)** cells were treated as described under Section “Materials and Methods” with vehicle alone **(A,C)** or 10 mM MβCD **(B,D)**. Cells were fixed in 3% PFA and treated with filipin (50 μg/mL) for 2 h at room temperature. Projection of each z-stack acquired through confocal microscopy images was merged with DIC images. Filipin labels free cholesterol present in the membranes (red arrow) and in the cytosol (white arrow). Scale bar: 10 μm, n, nucleus.

Similar cholesterol content was quantified in untreated MDA-MB-231 and -435 cells, with 11.1 ± 2.9 μg cholesterol/mg cell protein and 10.3 ± 2.9 μg cholesterol/mg cell protein, respectively. MDA-MB-231 and -435 cells were then treated with increasing concentrations of MβCD (5, 10, and 20 mM) to extract cholesterol and the effect on cell cholesterol depletion was measured (**Figure 2**). Five millimolar MβCD had no effect on cell cholesterol amount in the two cell lines. In MDA-MB-231 cells a depletion peak of cell cholesterol was observed at 10 mM of MβCD (**Figure 2A**). In contrast, in MDA-MB-435 cells cell cholesterol amount did not vary upon MβCD treatment (**Figure 2B**). Filipin-labeled cells revealed that cholesterol was predominantly distributed in cytosol for MDA-MB-231 cells (**Figure 3A**, white arrow) and in plasma membrane in MDA-MB-435 cells (**Figure 3C**, red arrow). Interestingly, efficient extraction of cholesterol upon MβCD treatment was observed in MDA-MB-231 cells (**Figure 3B**) but not in MDA-MB-435 cells (**Figure 3D**).

### Decrease of Cell Cholesterol Content Potentiates Shedding of LRP-1 without Affecting the Expression of LRP-1, MT1-MMP, and ADAM-12

**FIGURE 4:**
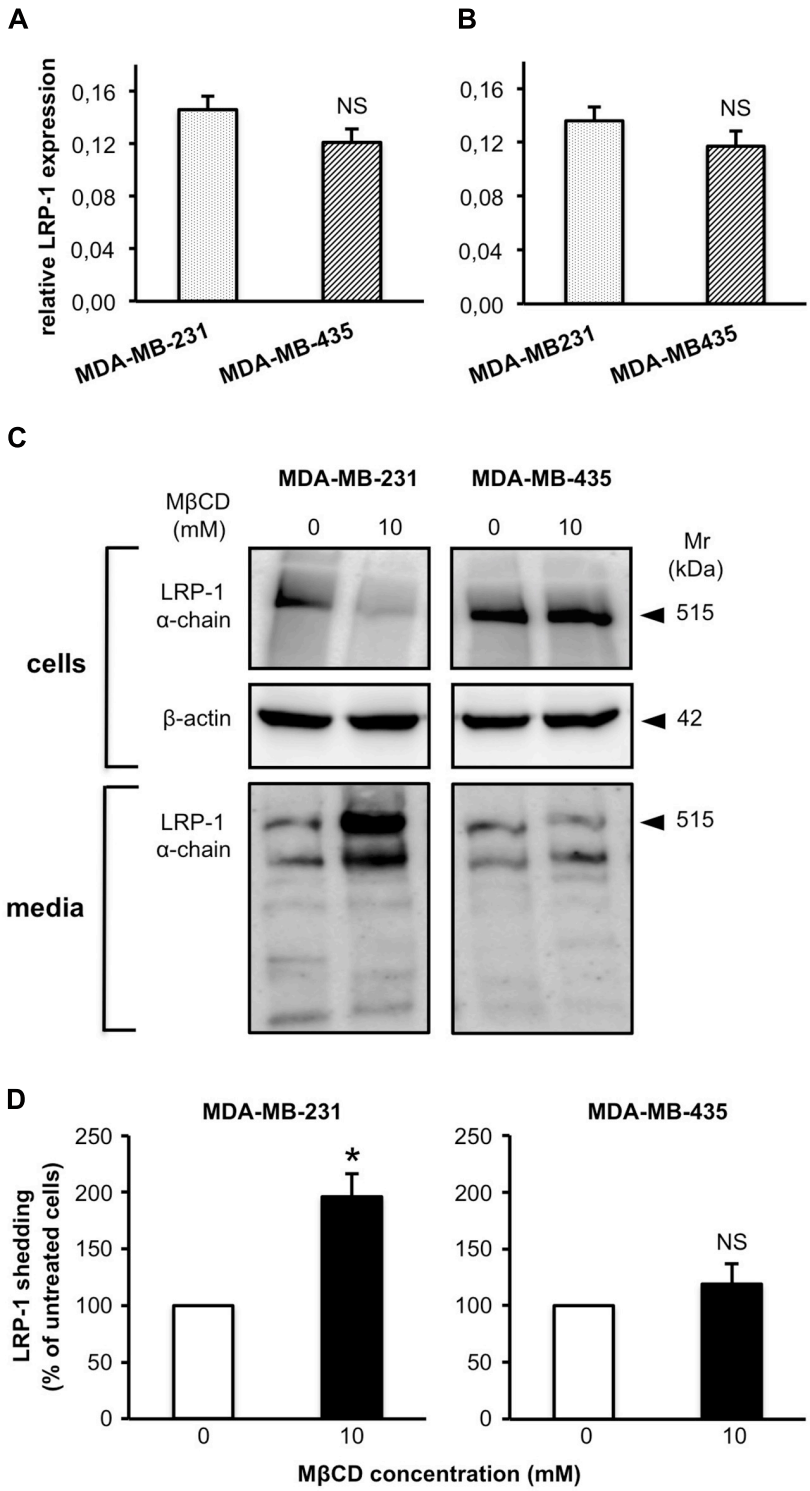
**Depletion of cellular cholesterol by MβCD increases shedding of the LRP-1 ectodomain.** MDA-MB-231 and MDA-MB-435 cells were treated as described under Section “Materials and Methods” with vehicle alone or 10 mM MβCD. Basal level expression of LRP-1 was measured by quantitative real-time PCR. Data were normalized to ribosomal proteins RPL32 **(A)** and RS18 **(B)**. Western blotting of LRP-1 α-chain and β-actin was performed from cell lysates, and blotting of LRP-1 α-chain in corresponding amounts of 24-h conditioned medium. Data are from a representative experiment **(C)**. LRP-1 ectodomain shedding was quantified on Western blots of LRP-1 α-chain released in the concentrated conditioned medium **(D)**. NS, not significant, ^∗^*P* < 0.05; Student’s *t*-test.

We then investigated if modulation of cell cholesterol amount by MβCD treatment had an impact on LRP-1 shedding process in MDA-MB-231 and -435 cells (**Figure 4**), as previously reported for the human fibrosarcoma HT1080 cells (Selvais et al., 2011). In the absence of MβCD treatment, LRP-1 expression is similar in MDA-MB-231 and -435 cells (**Figures 4A,B**). LRP-1 levels are also comparable in CHAPS extracts from MDA-MB-231 and -435 cells as well as in their respective conditioned media (**Figure 4C**). By using MβCD at 10 mM, a concentration that efficiently depleted MDA-MB-231 cells in cholesterol (**Figure 2A**), we observed a large decrease of LRP-1 in the CHAPS extracts of MDA-MB-231 cells that was accompanied by a twofold increase of soluble LRP-1 in conditioned media (**Figures 4C,D**). In contrast MβCD treatment, which did not modify cholesterol amount in MDA-MB-435 cells (**Figure 2B**), had no effect on LRP-1 shedding in these cells (**Figures 4C,D**).

**FIGURE 5:**
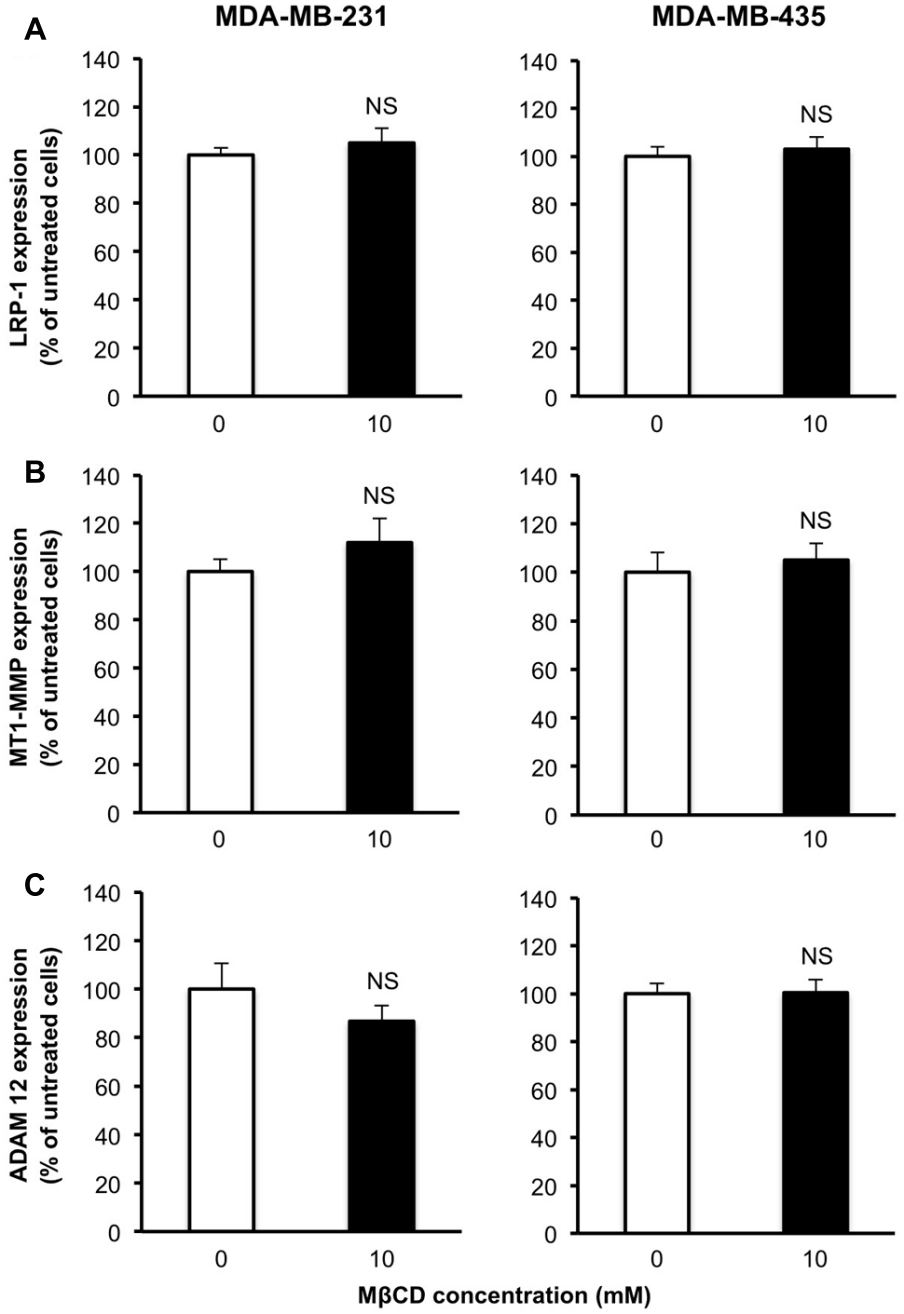
**Depletion of cellular cholesterol by MβCD has no effect on the expression of LRP-1 or its sheddases MT1-MMP and ADAM-12.** Quantitative real-time PCR of LRP-1 **(A)**, MT1-MMP **(B)** and ADAM-12 **(C)** mRNA levels. After normalization to β-actin mRNA amounts (Langlois et al., 2010), data were presented as a percentage of untreated cells. Values are mean ± SD (*n* = 3 for each cell line). NS, not significant; Student’s *t*-test.

To exclude that differences of LRP-1 shedding levels that we observed between the two breast cancer cell lines could be attributed to modulations of LRP-1, MT1-MMP and/or ADAM-12, its main sheddases (Selvais et al., 2011), we tested the effect of MβCD treatment on the expression of these three molecules. Neither LRP-1 mRNA (**Figure 5A**) nor MT1-MMP mRNA (**Figure 5B**) and ADAM-12 (**Figure 5C**) levels were affected by 10 mM MβCD treatment.

## Discussion

In the present study we investigated the relationship between cholesterol cell distribution and LRP-1 shedding efficiency. For this purpose, we used MDA-MB-231 and MDA-MB-435 cells, two cancer cell lines recently described for exhibiting different patterns of cholesterol localization, respectively in the cytoplasm and in the plasma membrane (Nieva et al., 2012). Treatment by MβCD decreased amount of cholesterol that was mainly localized in cytoplasm and stimulated removal of cell-surface LRP-1. In contrast, such a treatment had no effect on cholesterol levels predominantly distributed at the plasma membrane and on the release of the LRP-1 ectodomain. These discrepancies are not related to modified expression of LRP-1 and/or of its sheddases, MT1-MMP and ADAM-12. Altogether, these data suggested that cell distribution of cholesterol affects the shedding of LRP-1 from the cell surface.

The lipid profiling of MDA-MB-231 and MDA-MB-435 breast cancer cells was first investigated using Raman microspectroscopy. Raman microspectroscopy was performed on single living cells. The non-destructive and label free spectral analysis permitted to highlight the lipid contribution of the cytoplasmic compartment as a distinctive biochemical characteristic between the two cell types. The discriminant potential of this biophotonic approach was shown by a standard PCA. This unsupervised processing revealed also a marked intra-group variability as visible on the score plot of **Figure 1**. The origin of this variability could be investigated by carrying out spectral imaging at the cellular level (Abramczyk et al., 2015). Innovative devices, based on stimulate Raman scattering, have been recently proven to map the cellular lipid distribution in video-rate imaging (Ramachandran et al., 2015). Raman microspectroscopy allowed us to partially discriminate the two cell lines on the basis of their cytoplasmic spectral signature of lipids, including cholesterol as previously described (Nieva et al., 2012). Recent data obtained by fluorescence microscopy study after filipin staining indicated that cholesterol was mainly concentrated in cytoplasm of MDA-MB-231 cells while it was mostly distributed in plasma membrane of MDA-MB-435 cells (Nieva et al., 2012). In the present study, the observation of filipin-stained MDA-MB-231 and MDA-MB-435 cells by confocal microscope confirmed such findings.

The efficient depletion of cytoplasmic cholesterol in MDA-MB-231 cells after treatment with MβCD indicates that such a compound can pass through plasma membrane for extracting cholesterol from membranes of cytosolic vesicles, as previously reported for removal of lysosomal cholesterol in skin fibroblasts (Swaroop et al., 2012). Unability of MβCD to extract cholesterol mainly distributed in plasma membrane of MDA-MB-435 cells (**Figures 2** and **3**) is rather surprising. MβCD treatment has indeed been often associated with lipid raft disintegration (Zimina et al., 2005). The lipid rafts result of the interaction between cholesterol with sphingolipids in the outer exoplasmic leaflet of the lipid bilayer of cellular membranes. Cholesterol also interacts with phospholipids in the inner cytoplasmic leaflet of the lipid bilayer. Lipid rafts are considered to be present as a liquid-ordered phase while phospholipid-rich domains are in a disordered state (Simons and Ehehalt, 2002). Giant plasma-membrane vesicles represent a valuable physiological tool to investigate lipid phase separation (Baumgart et al., 2007). Using this model, Levental et al. (2009) demonstrated a cholesterol dependence of phase separation in complex membranes at physiological conditions. Moreover, using the same experimental model Sanchez et al. (2011) demonstrated that MβCD preferentially removed cholesterol from a liquid disordered phase. A computational microscopy study recently confirmed that cholesterol was preferentially extracted from the disordered regions compared to liquid-ordered domains of lipid model membranes (López et al., 2013). Altogether, these data suggest that MβCD-resistant cholesterol in membranes of the MDA-MB-435 cells reflects their richness in lipid rafts. This will be evaluated in a future experiment by atomic force microscopy, as recently proposed (Cremona et al., 2015).

The increase of transmembrane receptor shedding was often related to decrease of cell cholesterol amount, possibly by disintegration of lipid rafts and dynamic interactions of the sheddase and its target (Matthews et al., 2003; von Tresckow et al., 2004; Zimina et al., 2005). We showed similar correlation of increase of LRP-1 shedding by MT1-MMP and cell cholesterol decrease upon MβCD treatment in HT1080 cells (Selvais et al., 2011). Interestingly, our present study highlights a relationship between cholesterol cell distribution and LRP-1 shedding efficiency. Fluorescence imaging in living CHO cells clearly evidenced that intracellular cholesterol is mainly distributed in the endocytic recycling compartment and the *trans*-Golgi network (Mukherjee et al., 1998). However, the multiplicity of cholesterol transport systems makes difficult the establishment of specific trafficking route (Chang et al., 2006). Previous studies demonstrate that cholesterol intracellular trafficking and distribution, rather than total cholesterol levels, are regulatory factors in the β-amyloid precursor protein processing (Marzolo and Bu, 2009). Malnar et al. (2012) proposed that cholesterol regulates the β-amyloid precursor protein processing by modulating APP expression at the cell surface. To our knowledge, no relationship between intracellular cholesterol distribution and LRP-1 localization has been proposed so far.

## Conclusion

Our data suggest that intracellular cholesterol depletion may increase intracellular trafficking to cell surface of newly synthesized LRP-1 and/or recycled LRP-1 after endocytosis process. Consequently, enhancement of LRP-1 shedding upon cholesterol depletion should reflect a higher disponibility of the sheddase substrate, i.e., LRP-1, at the cell surface. However, the question whether intracellular cholesterol depletion has an impact on LRP-1 localization remains to be elucidated.

## Author Contributions

BD, AW, HS, MF, J-FA contributed to the acquisition and analysis of data for the work; SD, OP, and JD contributed to the conception, design of the work and to the analysis and interpretation of data for the work; HE contributed to the conception, design of the work and to the analysis and interpretation of data for the work and written the manuscript.

## Conflict of Interest Statement

The authors declare that the research was conducted in the absence of any commercial or financial relationships that could be construed as a potential conflict of interest.
